# Parenterally Administered Norovirus GII.4 Virus-Like Particle Vaccine Formulated with Aluminum Hydroxide or Monophosphoryl Lipid A Adjuvants Induces Systemic but Not Mucosal Immune Responses in Mice

**DOI:** 10.1155/2018/3487095

**Published:** 2018-02-28

**Authors:** Suvi Heinimäki, Maria Malm, Timo Vesikari, Vesna Blazevic

**Affiliations:** Vaccine Research Center, University of Tampere, Tampere, Finland

## Abstract

Norovirus (NoV) is a main cause of acute gastroenteritis across all ages worldwide. NoV vaccine candidates currently in clinical trials are based on noninfectious highly immunogenic virus-like particles (VLPs) delivered intramuscularly (IM). Since NoV is an enteric pathogen, it is likely that mucosal immunity has a significant role in protection from infection in the intestine. Due to the fact that IM delivery of NoV VLPs does not generate mucosal immunity, we investigated whether NoV genotype GII.4 VLPs coadministered with aluminum hydroxide (Al(OH)_3_) or monophosphoryl lipid A (MPLA) would induce mucosal antibodies in mice. Systemic as well as mucosal IgG and IgA antibodies in serum and intestinal and nasal secretions were measured. As expected, strong serum IgG, IgG1, and IgG2a antibodies as well as a dose sparing effect were induced by both Al(OH)_3_ and MPLA, but no mucosal IgA antibodies were detected. In contrast, IN immunization with GII.4 VLPs without an adjuvant induced systemic as well as mucosal IgA antibody response. These results indicate that mucosal delivery of NoV VLPs is needed for induction of mucosal responses.

## 1. Introduction

The need for norovirus (NoV) vaccine is apparent, as NoV is the most common cause of acute viral gastroenteritis worldwide with approximately 200,000 annual deaths [1]. It infects humans of all ages, but children <5 years of age, the elderly, and immunocompromised individuals are at the highest risk. The most advanced NoV vaccine in phase II clinical trials is based on virus-like particles (VLPs) administered intramuscularly (IM) with aluminum hydroxide (Al(OH)_3_) [2] or a combination of Al(OH)_3_ and monophosphoryl lipid A (MPLA) [3–6]. Alternative intranasal (IN) administration of NoV VLPs has previously been evaluated as well [7, 8]. Despite the lack of the definite vaccine-associated correlate of protection for NoV, mucosal immunity is known to play a significant role in protection from infection and disease caused by enteric pathogens, including NoV [9–12].

At present, there are only a few adjuvants approved for human use, and none for mucosal delivery [13, 14]. Aluminum salts (Alum) and MPLA are adjuvants commonly included in the formulation of licensed protein subunit vaccines, such as VLP-based vaccines against human papilloma virus (Cervarix®, Gardasil®) and hepatitis B virus (Engerix-B®, Recombivax HB®). Alum, the first and predominant adjuvant in human vaccines, is employed to stabilize the vaccine antigen and also as a delivery system [13, 15]. MPLA, a new-generation toll-like receptor- (TLR-) based adjuvant, is a TLR4 agonist, which activates innate immunity [16, 17], thereby influencing the development of adaptive immunity. Recently, alum has been described to possess immunomodulatory features as well [18]. Both of these adjuvants stimulate systemic immune responses, when administered parenterally with the vaccine antigens. However, their effect on antigen-specific mucosal immunity is not known.

We have recently shown that NoV GII.4 VLPs induce protective IgA antibodies in mucosal lavages of mice immunized via intranasal (IN), but not IM, route [9]. Here, we investigated if IM delivery of NoV GII.4 VLPs formulated with commonly used adjuvants, Al(OH)_3_ or MPLA, has an effect on generation of NoV-specific mucosal immunity.

## 2. Materials and Methods

### 2.1. Recombinant NoV VLP Production

NoV GII.4-1999 (reference strain accession number AF080551) VLPs used for immunization and as antigen in immunological assays were produced by recombinant baculovirus technology in Sf9 insect cells and purified as described in detail elsewhere [19].

### 2.2. Immunization and Sample Preparation

Female 7-week-old BALB/c OlaHsd mice (5–8 mice/experimental group) (Envigo, Horst, the Netherlands) were immunized IM two times (at study weeks 0 and 3) with 0.3 *μ*g dose of GII.4 VLPs alone or formulated with 100 *μ*g of Al(OH)_3_ (Alhydrogel; InvivoGen, San Diego, CA) or 5 *μ*g of MPLA from *S. minnesota R595* (InvivoGen). In addition, two groups of mice received a combination vaccine [20] containing 10 *μ*g GII.4 VLPs via IM and IN delivery. Mice administered IM or IN with a carrier (sterile PBS only) served as control groups. Immunizations were performed under general anesthesia induced with a mixture of ketamine (Ketalar®; Pfizer Ltd., NY) and medetomidine (Dorbene®; Syva, Leon, Spain).

To test the kinetics of the antibody responses in sera, tail blood samples (diluted 1 : 200 in PBS at the time of collection) were collected at study weeks 0 (prebleed, nonimmune sera) and 3. Mice were sacrificed at study week 5 by decapitation, when whole blood, intestinal secretions (feces), nasal washes (NWs), and mesenteric lymph nodes (MLNs) were collected. Preparation of blood samples and lymphoid tissues was conducted according to the previously published procedures [21], except a single-cell suspension from group-wise pooled MLNs was prepared without a lysis step of red blood cells. Fecal pellets and NWs were processed as previously published [9, 22]. All of the experimental procedures conducted were in accordance with the regulations and guidelines of the Finnish National Experiment Board.

### 2.3. Detection of NoV-Specific Serum and Mucosal Antibodies by ELISA

Serum samples of individual mice were serially diluted two-fold from 1 : 200 (for IgG) or 1 : 20 (for IgA) and tested in ELISA for the presence of NoV GII.4-specific IgG, IgG1, IgG2a, and IgA antibodies as described elsewhere [10, 21]. Fecal suspensions (10%) and NWs were two-fold serially diluted from 1 : 5 and studied for IgG and IgA antibodies. Briefly, 96-well half-area polystyrene plates (Corning Inc., Corning, NY) were coated with 50 ng of GII.4 VLPs per well. Sample dilutions were added on the plates, and the bound antibodies were detected with horseradish peroxidase- (HRP-) conjugated anti-mouse IgG (Sigma-Aldrich, St. Louis, MO), IgG1 (Invitrogen, Carlsbad, CA), IgG2a (Invitrogen) or IgA (Sigma-Aldrich), and SIGMAFAST OPD substrate (Sigma-Aldrich). Optical density (OD) values at 490 nm were measured by a microplate reader (Victor^2^ 1420; PerkinElmer, Waltham, MA). A sample was considered positive if the OD_490_ was above the cut-off value (mean OD_490_ + 3 × SD of the control mice and OD_490_ > 0.1). The end-point titer was defined as the reciprocal of the highest dilution with an OD_490_ above the cut-off value. For negative samples with the OD_490_ below the cut-off limit, a half of the starting dilution was assigned for the titer.

### 2.4. Detection of NoV-Specific Antibody Secretion by MLN Cells

Group-wise pooled MLN cells (4 × 10^6^ cell/ml) from immunized mice were stimulated *in vitro* [23, 24] with 5 *μ*g/ml of GII.4 VLPs or culture medium (RPMI 1640 supplemented with 10% FBS, 100 U/ml penicillin, 100 *μ*g/ml streptomycin, 50 *μ*M 2-mercaptoethanol, and 2 mM L-glutamine; all from Sigma-Aldrich) only. After incubation at 37°C for 10 days, supernatants were collected and stored at −20°C until analyzed in ELISA for anti-GII.4 IgG and IgA antibodies as described above.

### 2.5. Statistical Analyses

Fisher's exact test was employed to assess the intergroup differences in the IgG and IgA end-point titers. The Mann–Whitney *U* test and Kruskal-Wallis test were used to compare differences between the nonparametric observations of two or more independent groups. All analyses were conducted by IBM SPSS Statistics for Windows Version 23.0 (IBM Corp., Armonk, NY). The statistically significant difference was defined as *p* ≤ 0.05.

## 3. Results

### 3.1. Induction of NoV GII.4-Specific Serum IgG and IgA Antibodies

**Figure 1:**
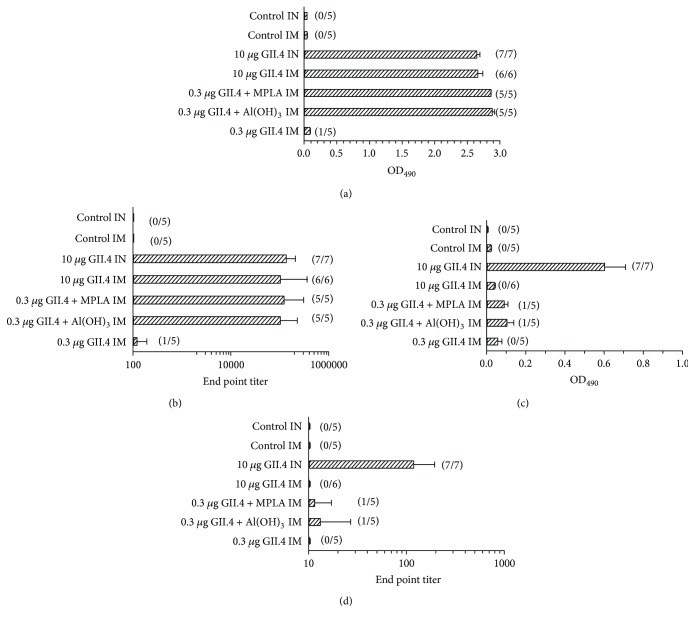
NoV GII.4-specific systemic IgG (a, b) and IgA (c, d) antibody responses after IM immunization with 0.3 or 10 *μ*g of NoV GII.4 VLPs alone or 0.3 *μ*g of VLPs combined with Al(OH)_3_ or MPLA, or IN immunization with 10 *μ*g of VLPs alone. Group mean OD_490_ values with standard error of the means of IgG (a) and IgA (c) antibodies in 1 : 200 (a) and 1 : 20 (c) diluted sera of experimental mice. Control mice received PBS only. End-point titers of IgG (b) and IgA (d) antibodies in sera of the experimental groups. Bars represent reciprocal of log_10_ geometric mean titers with 95% confidence intervals. The number of positive/tested mice is denoted on each figure in parenthesis.

Effect of two commonly used adjuvants on NoV GII.4-specific serum IgG and IgA antibody responses was investigated by immunizing the experimental mice twice IM with 0.3 or 10 *μ*g of GII.4 VLPs alone or 0.3 *μ*g dose combined with Al(OH)_3_ or MPLA. For comparison, one group of mice received 10 *μ*g GII.4 VLPs via IN delivery. IM immunization with 0.3 *μ*g of GII.4 VLPs did not elicit a significant serum IgG response (geometric mean titer, GMT = 119; 95% CI = 74–192), whereas coadministration of 0.3 *μ*g VLPs with Al(OH)_3_ or MPLA resulted in robust (GMTs > 5log10) NoV GII.4-specific IgG levels (Figures 1(a) and 1(b)). Also, IM and IN administration of 10 *μ*g dose of VLPs induced high levels of anti-GII.4 IgG antibodies (Figures 1(a) and 1(b)). No significant difference (*p* = 0.87) was observed in the magnitude of the responses induced by 0.3 *μ*g dose of VLPs with either of the adjuvants and 10 *μ*g dose of VLPs via IM or IN route.

Parenteral delivery of VLPs without an adjuvant did not elicit detectable serum anti-NoV IgA antibodies, but very low serum IgA was observed in 1/5 mice after immunizations with 0.3 *μ*g dose formulated with Al(OH)_3_ (GMT = 13; 95% CI = 6–27) or MPLA (GMT = 11; 95% CI = 8–17) (Figures 1(c) and 1(d)). In contrast, IN administration of the GII.4 VLPs alone generated a significantly higher (*p* = 0.006) IgA response (GMT = 119; 95% CI = 73–194) compared with IM administration of the adjuvanted VLP formulations (Figures 1(c) and 1(d)). No GII.4-specific IgG or IgA antibodies were detected in sera of control mice (Figures 1(a)–1(d)).

### 3.2. Kinetics and Th1/Th2 Dichotomy Induced by Al(OH)_3_ and MPLA

**Figure 2:**
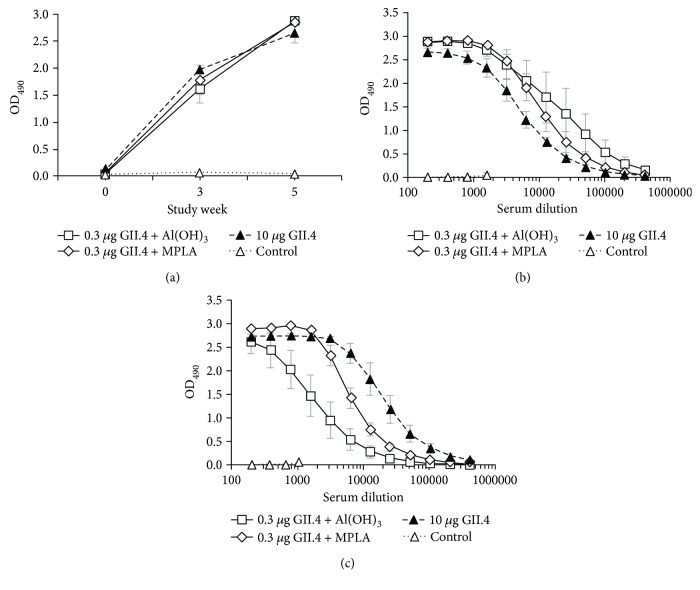
Development of IgG (a) and IgG subtype antibodies (b, c) in mice immunized IM with 0.3 *μ*g of GII.4 VLPs formulated with Al(OH)_3_ or MPLA or 10 *μ*g of GII.4 VLPs alone. (a) Kinetics of NoV GII.4-specific total IgG antibodies in sera of mice immunized with the antigenic formulations at study weeks 0 and 3. Control mice received PBS only. Group mean OD_490_ values with standard error of the means of individual tail blood samples collected at indicated study weeks and termination sera at week 5 are shown. End-point titrations of anti-GII.4 IgG1 (b) and IgG2a (c) antibodies in termination sera at week 5. Mean titration curves with standard errors of the mean of the experimental groups are shown.

To study the effect of Al(OH)_3_ and MPLA on kinetics of serum NoV GII.4-specific antibody responses, 1 : 200 diluted sera from mice immunized IM on a two-dose schedule at an interval of three weeks were tested for IgG antibodies. After the first immunization, 0.3 *μ*g dose of GII.4 VLPs formulated with Al(OH)_3_ or MPLA as well as 10 *μ*g dose of VLPs alone resulted in comparable IgG responses (*p* = 0.679) (Figure 2(a)). The second dose of these antigenic formulations delivered at week 3 enhanced the already established strong responses in all experimental groups (*p* = 0.176), as observed at week 5 (Figure 2(a)). Control mice remained negative for GII.4-specific IgG (OD_490_ < 0.1) during the study period (Figure 2(a)).

Determination of IgG subtype titers showed generation of both IgG1 (a marker of a Th2-type response) (Figure 2(b)) and IgG2a (a marker of a Th1 type response) (Figure 2(c)) antibodies by both adjuvanted GII.4 VLP formulations. No statistical difference was detected in the IgG1 titers (*p* = 0.122) induced by 0.3 *μ*g of VLPs in the presence of Al(OH)_3_ or MPLA or 10 *μ*g of VLPs alone (Figure 2(b)). In contrast, IgG2a titers differed between the experimental groups (Figure 2(c)), Al(OH)_3_ adjuvanted group having significantly lower titers compared to other groups (*p* = 0.039). End-point titer IgG1/IgG2a (Th2/Th1) ratio was 2 : 1 for mice immunized with a combination of VLPs and MPLA and 10 : 1 for VLPs and Al(OH)_3_. No GII.4-specific IgG subtype antibodies were detected in sera of control mice (Figures 2(b) and 2(c)).

### 3.3. Induction of NoV GII.4-Specific Antibodies in Mucosal Secretions

**Figure 3:**
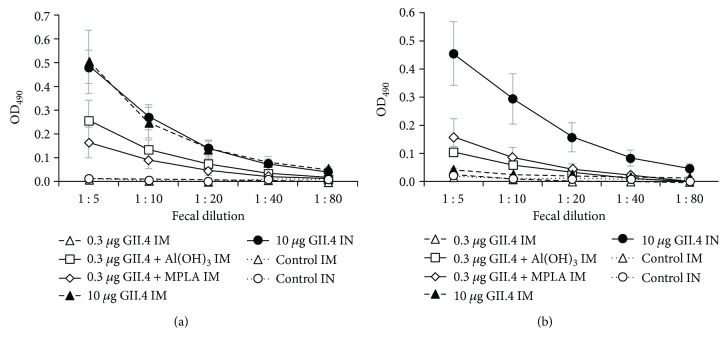
NoV GII.4-specific fecal antibody responses after IM immunization with 0.3 or 10 *μ*g of NoV GII.4 VLPs alone or 0.3 *μ*g of VLPs combined with Al(OH)_3_ or MPLA, or IN immunization with 10 *μ*g of VLPs alone. End-point titrations of anti-GII.4 IgG (a) and IgA (b) antibodies in 10% fecal suspensions of experimental mice. Control mice received PBS only. Mean titration curves with standard errors of the mean of the experimental groups are shown.

In order to investigate if NoV VLPs coadministered with Al(OH)_3_ or MPLA induced antibodies at mucosal surfaces, 10% fecal suspensions and NW samples of experimental animals were tested for the presence of anti-GII.4 IgG and IgA antibodies. As expected, mice immunized IM with 0.3 *μ*g dose of GII.4 VLPs alone did not develop intestinal IgG antibodies (Figure 3(a)). Instead, all other experimental groups had similar levels of IgG (*p* = 0.277) in the intestines (Figure 3(a)).

IM delivery of VLPs in the absence of adjuvants did not generate intestinal IgA antibodies (GMT = 2.5), but fecal IgA response was detected in 1/5 and 2/5 mice after coadministration of 0.3 *μ*g dose with Al(OH)_3_ (GMT = 3; 95% CI = 2–7) or MPLA (GMT = 5; 95% CI = 2–15) (Figure 3(b)), corroborating serum IgA response (Figure 1(d)). Significantly greater level of IgA in the intestine (*p* = 0.023) was elicited by mucosal IN delivery of 10 *μ*g of VLPs (GMT = 25; 95% CI = 12–53) (Figure 3(b)).

**Figure 4:**
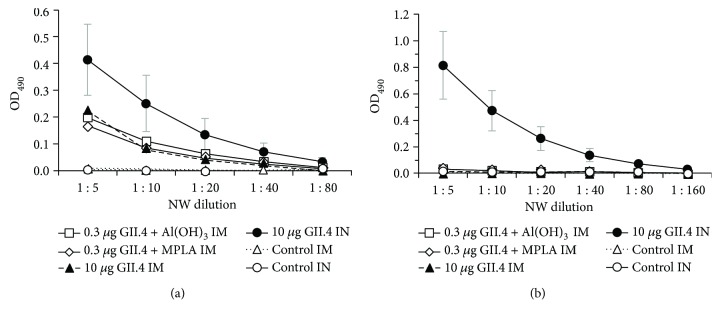
Mucosal antibody responses in nasal washes after IM immunization with 0.3 *μ*g of NoV GII.4 VLPs formulated with Al(OH)_3_ or MPLA, or IM or IN immunization with 10 *μ*g of VLPs alone. End-point titrations of anti-GII.4 IgG (a) and IgA (b) antibodies in nasal washes (NWs) of experimental mice. Control mice received PBS only. Mean titration curves with standard errors of the mean of the experimental groups are shown.

Nasal lavages from experimental groups with intestinal antibodies were also tested for the presence of GII.4-specific IgG and IgA antibodies. Similar to fecal specimens, comparable IgG levels (*p* = 0.902) were induced by 0.3 *μ*g of VLPs in the presence of Al(OH)_3_ (GMT = 6; 95% CI = 2–15) or MPLA (GMT = 6; 95% CI = 4–8) via IM delivery, or by 10 *μ*g of VLPs alone via IM (GMT = 7; 95% CI = 3–16) or IN delivery (GMT = 12; 95% CI = 4–33) (Figure 4(a)). In contrast, only IN administration of VLPs resulted in generation of IgA antibodies (GMT = 30; 95% CI = 9–102) in the nasal secretions (Figure 4(b)).

### 3.4. Al(OH)_3_ or MPLA Induced No Production of IgA Antibodies by MLN Cells

**Figure 5:**
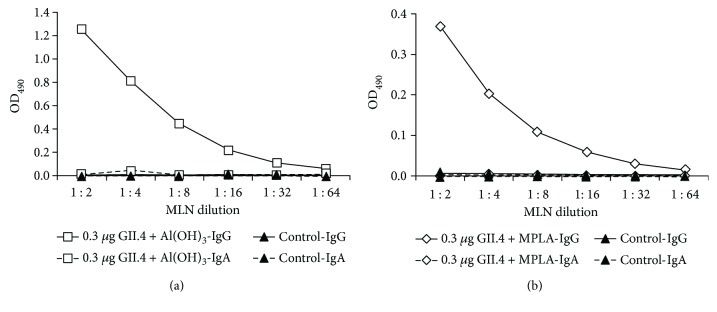
IgG and IgA antibodies in cells from mesenteric lymph nodes after IM immunization with 0.3 *μ*g of NoV GII.4 VLPs formulated with Al(OH)_3_ (a) or MPLA (b). End-point titration curves of anti-GII.4 IgG and IgA antibodies in cell culture supernatants from pooled mesenteric lymph nodes (MLNs) of experimental groups stimulated *in vitro* with 5 *μ*g GII.4 VLPs. Control mice received PBS only.

In order to confirm a lack of mucosal IgA antibodies induced by the two adjuvants in mucosal secretions (Figures 3 and 4), MLN cells from Al(OH)_3_ and MPLA immunized mice were tested for the production of IgG and IgA antibodies. Cells from mice receiving VLPs in the presence of Al(OH)_3_ or MPLA via IM delivery responded with considerable IgG production to *in vitro* stimulation with GII.4 VLPs (Figures 5(a) and 5(b)). On the contrary, neither of the adjuvanted formulations induced IgA production by MLN cells (Figures 5(a) and 5(b)), indicating that MLNs were not inductive sites of IgA responses detected in the intestinal secretions after IM immunization. No anti-GII.4 IgG or IgA production was detected when MLN cells were stimulated with the culture medium only (data not shown).

## 4. Discussion

Protection against pathogens at mucosal surfaces is largely dependent on secretory IgA effectively induced by IN immunization [25, 26]. Because of NoV transmission through intestinal mucosa, induction of mucosal immunity to NoV is likely a pivotal factor to be taken into consideration in NoV vaccine development. Although parenteral immunization routes are not generally considered potent inducers of mucosal immunity [9, 10, 27–29], we investigated if mucosal antibodies are induced by IM immunization of BALB/c mice with NoV GII.4 VLPs formulated with the widely used systemic adjuvants Al(OH)_3_, a gold standard delivery system, or MPLA, a TLR4 agonist. It has been recently demonstrated that TLR ligands (TLR3/TLR4) alter migration patterns of dendritic cells *in vivo* and promote induction of mucosal responses to codelivered antigens as a consequence of antigen-loaded dendritic cells migrating to both draining and nondraining lymph nodes [30]. Therefore, we also investigated if IM immunization of NoV VLP antigens codelivered with these adjuvants causes lymphoid cell dissemination to remote sites like MLN.

The current NoV VLP vaccine candidate in the most advanced phases of clinical trials is administered IM in a formulation with MPLA and/or Al(OH)_3_, being effective in inducing high systemic antibody responses [3–6]. After the challenge, protection was seen against moderate to severe forms of acute gastroenteritis, without significant reductions in NoV shedding [6]. Mucosal responses, namely, antibody-secreting cells (ASCs) with mucosal homing phenotype, derived from a recall of the mucosal response primed by prior exposures to NoV may be responsible for the observed protection [31]. In gnotobiotic calves, fecal IgA-mediated protection from virulent bovine NoV challenge was demonstrated only after mucosal (IN) immunization with bovine NoV VLPs [32]. Several other reports have also shown importance of mucosal immunity, especially IgA, in protection from NoV infection [9, 11, 12]. Similarly, parenterally delivered inactivated polio vaccine (IPV) generates protective serum antibodies but not local intestinal mucosal antibodies in the gut, therefore being sufficient to protect against disease but insufficient to prevent wild poliovirus from replicating in intestines and spreading to the environment [33, 34].

In this study, high levels of serum IgG antibodies were induced by both adjuvants. Al(OH)_3_ and MPLA promoted >30-fold dose sparing above nonadjuvanted dose (0.3 *μ*g versus 10 *μ*g dose, resp.) and induced considerable levels of IgG1, a marker of Th2 response, as well as IgG2a, a marker of Th1 response. Although MPLA promotes a Th1 bias [17, 35], our data showed induction of a balanced Th1/Th2-type response by this adjuvant. In contrast, Al(OH)_3_ stimulated Th2-biased response, which is in concordance with previous observations [13, 36, 37]. A biased response depends on several factors such as the route of delivery, animal strain, and the vaccine antigen used, thereby explaining apparent discrepancies of our results. Despite different mechanisms of the two adjuvants employed in the present study, one being primarily a delivery or depot system and another an immunostimulator [13], our data indicate that Al(OH)_3_ and MPLA work similarly for IM delivered NoV VLPs in terms of the dose sparing, kinetics, and generation of systemic IgG antibodies.

Our recent study demonstrated that IgA levels in mucosal tissues correlated with blocking activity, suggesting that IgA, but not IgG, was the main antibody neutralizing NoV on the mucosal surfaces [9]. In concordance with [9], current results show that mucosal IgA antibodies were induced by IN, but not IM, administration of NoV VLPs without an adjuvant. Only low level of fecal IgA was detected in a few mice immunized IM with NoV GII.4 VLPs and either of the adjuvants. Because of the similarly low IgA levels in sera of these animals, the detected antibody is likely a systemic IgA passively exuded from serum to mucosal secretions. In order to ensure that no NoV GII.4-specific secretory IgA response in mucosa was missed, we also tested NWs and vaginal washes (data not shown) for the presence of IgA antibodies. Only IN delivery of GII.4 NoV VLPs induced IgA antibodies in these secretions. In addition, in support to the serum origin of IgA, no IgA ASCs were detected in MLN cells of mice immunized with NoV VLP vaccine formulated with Al(OH)_3_ or MPLA adjuvant. Similarly, Bessa and colleagues have detected IgG, but not IgA, ASCs in MLN after subcutaneous immunization of mice with a vaccine platform based on VLPs [38].

Our results clearly demonstrate the lack of the mucosal IgA antibody generation in naïve animals after IM delivery of NoV VLPs regardless of the adjuvants Al(OH)_3_ or MPLA used. Others have shown that parenteral delivery (IM and intradermal) of vaccine antigens with mucosal adjuvant dmLT (a detoxified form of the heat-labile enterotoxin of *E. coli*) promotes mucosal immunity [24], likely by inducing mucosal homing receptors on B cells and their migration to mucosal compartments [39], although the exact mechanism is not completely understood. Therefore, the field of NoV vaccine development should consider mucosal delivery of NoV VLP vaccines [40], or at least inclusion of mucosal adjuvants or components targeted to mucosal trafficking, to immunize naïve individuals, such as infants and young children, to ensure induction of mucosal responses in the gut, the port of entry for enteric viruses like NoV.

## 5. Conclusions

The present study demonstrates that IM administration of NoV GII.4 VLPs formulated with Al(OH)_3_ or MPLA does not induce significant mucosal IgA antibodies in mice. Instead, IN immunization with GII.4 VLPs alone elicited NoV-specific mucosal immunity. These results underline the importance of mucosal delivery route in induction of potent mucosal responses.

## References

[1] Patel M. M., Widdowson M. A., Glass R. I., Akazawa K., Vinje J., Parashar U. D. (2008). Systematic literature review of role of noroviruses in sporadic gastroenteritis. *Emerging Infectious Diseases*.

[2] Baehner F., Bogaerts H., Goodwin R. (2016). Vaccines against norovirus: state of the art trials in children and adults. *Clinical Microbiology and Infection*.

[3] Treanor J. J., Atmar R. L., Frey S. E. (2014). A novel intramuscular bivalent norovirus virus-like particle vaccine candidate—reactogenicity, safety, and immunogenicity in a phase 1 trial in healthy adults. *The Journal of Infectious Diseases*.

[4] Ramani S., Neill F. H., Ferreira J. (2017). B-cell responses to intramuscular administration of a bivalent virus-like particle human norovirus vaccine. *Clinical and Vaccine Immunology*.

[5] Atmar R. L., Baehner F., Cramer J. P. (2016). Rapid responses to 2 virus-like particle norovirus vaccine candidate formulations in healthy adults: a randomized controlled trial. *The Journal of Infectious Diseases*.

[6] Bernstein D. I., Atmar R. L., Lyon G. M. (2015). Norovirus vaccine against experimental human GII.4 virus illness: a challenge study in healthy adults. *The Journal of Infectious Diseases*.

[7] Atmar R. L., Bernstein D. I., Harro C. D. (2011). Norovirus vaccine against experimental human Norwalk virus illness. *The New England Journal of Medicine*.

[8] El-Kamary S. S., Pasetti M. F., Mendelman P. M. (2010). Adjuvanted intranasal Norwalk virus-like particle vaccine elicits antibodies and antibody-secreting cells that express homing receptors for mucosal and peripheral lymphoid tissues. *The Journal of Infectious Diseases*.

[9] Tamminen K., Malm M., Vesikari T., Blazevic V. (2016). Mucosal antibodies induced by intranasal but not intramuscular immunization block norovirus GII.4 virus-like particle receptor binding. *Viral Immunology*.

[10] Malm M., Tamminen K., Vesikari T., Blazevic V. (2015). Comparison of intramuscular, intranasal and combined administration of norovirus virus-like particle subunit vaccine candidate for induction of protective immune responses in mice. *Journal of Clinical & Cellular Immunology*.

[11] Lindesmith L., Moe C., Marionneau S. (2003). Human susceptibility and resistance to Norwalk virus infection. *Nature Medicine*.

[12] Ramani S., Neill F. H., Opekun A. R. (2015). Mucosal and cellular immune responses to Norwalk virus. *The Journal of Infectious Diseases*.

[13] Apostolico Jde S., Lunardelli V. A., Coirada F. C., Boscardin S. B., Rosa D. S. (2016). Adjuvants: classification, modus operandi, and licensing. *Journal of Immunology Research*.

[14] Lycke N. (2012). Recent progress in mucosal vaccine development: potential and limitations. *Nature Reviews Immunology*.

[15] Ghimire T. R., Benson R. A., Garside P., Brewer J. M. (2012). Alum increases antigen uptake, reduces antigen degradation and sustains antigen presentation by DCs *in vitro*. *Immunology Letters*.

[16] Mata-Haro V., Cekic C., Martin M., Chilton P. M., Casella C. R., Mitchell T. C. (2007). The vaccine adjuvant monophosphoryl lipid a as a TRIF-biased agonist of TLR4. *Science*.

[17] Casella C. R., Mitchell T. C. (2008). Putting endotoxin to work for us: monophosphoryl lipid a as a safe and effective vaccine adjuvant. *Cellular and Molecular Life Sciences*.

[18] Kool M., Petrilli V., De Smedt T. (2008). Cutting edge: alum adjuvant stimulates inflammatory dendritic cells through activation of the NALP3 inflammasome. *The Journal of Immunology*.

[19] Huhti L., Blazevic V., Nurminen K., Koho T., Hytönen V., Vesikari T. (2010). A comparison of methods for purification and concentration of norovirus GII-4 capsid virus-like particles. *Archives of Virology*.

[20] Tamminen K., Lappalainen S., Huhti L., Vesikari T., Blazevic V. (2013). Trivalent combination vaccine induces broad heterologous immune responses to norovirus and rotavirus in mice. *PLoS One*.

[21] Tamminen K., Huhti L., Koho T. (2012). A comparison of immunogenicity of norovirus GII-4 virus-like particles and P-particles. *Immunology*.

[22] Blazevic V., Lappalainen S., Nurminen K., Huhti L., Vesikari T. (2011). Norovirus VLPs and rotavirus VP6 protein as combined vaccine for childhood gastroenteritis. *Vaccine*.

[23] Elson C. O., Ealding W. (1984). Generalized systemic and mucosal immunity in mice after mucosal stimulation with cholera toxin. *The Journal of Immunology*.

[24] Norton E. B., Bauer D. L., Weldon W. C., Oberste M. S., Lawson L. B., Clements J. D. (2015). The novel adjuvant dmLT promotes dose sparing, mucosal immunity and longevity of antibody responses to the inactivated polio vaccine in a murine model. *Vaccine*.

[25] Lycke N. (1998). T cell and cytokine regulation of the IgA response. *Chemical Immunology*.

[26] Kozlowski P. A., Williams S. B., Lynch R. M. (2002). Differential induction of mucosal and systemic antibody responses in women after nasal, rectal, or vaginal immunization: influence of the menstrual cycle. *The Journal of Immunology*.

[27] Moldoveanu Z., Clements M. L., Prince S. J., Murphy B. R., Mestecky J. (1995). Human immune responses to influenza virus vaccines administered by systemic or mucosal routes. *Vaccine*.

[28] Sasaki S., Sumino K., Hamajima K. (1998). Induction of systemic and mucosal immune responses to human immunodeficiency virus type 1 by a DNA vaccine formulated with QS-21 saponin adjuvant via intramuscular and intranasal routes. *Journal of Virology*.

[29] Herremans T. M., Reimerink J. H., Buisman A. M., Kimman T. G., Koopmans M. P. (1999). Induction of mucosal immunity by inactivated poliovirus vaccine is dependent on previous mucosal contact with live virus. *The Journal of Immunology*.

[30] Enioutina E. Y., Bareyan D., Daynes R. A. (2008). TLR ligands that stimulate the metabolism of vitamin D3 in activated murine dendritic cells can function as effective mucosal adjuvants to subcutaneously administered vaccines. *Vaccine*.

[31] Sundararajan A., Sangster M. Y., Frey S. (2015). Robust mucosal-homing antibody-secreting B cell responses induced by intramuscular administration of adjuvanted bivalent human norovirus-like particle vaccine. *Vaccine*.

[32] Han M. G., Cheetham S., Azevedo M., Thomas C., Saif L. J. (2006). Immune responses to bovine norovirus-like particles with various adjuvants and analysis of protection in gnotobiotic calves. *Vaccine*.

[33] Hird T. R., Grassly N. C. (2012). Systematic review of mucosal immunity induced by oral and inactivated poliovirus vaccines against virus shedding following oral poliovirus challenge. *PLoS Pathogens*.

[34] Ogra P. L., Karzon D. T., Righthand F., MacGillivray M. (1968). Immunoglobulin response in serum and secretions after immunization with live and inactivated poliovaccine and natural infection. *The New England Journal of Medicine*.

[35] Vernacchio L., Bernstein H., Pelton S. (2002). Effect of monophosphoryl lipid A (MPL®) on T-helper cells when administered as an adjuvant with pneumocococcal–CRM_197_ conjugate vaccine in healthy toddlers. *Vaccine*.

[36] Brewer J. M. (2006). (How) do aluminium adjuvants work?. *Immunology Letters*.

[37] Lindblad E. B. (2004). Aluminium compounds for use in vaccines. *Immunology and Cell Biology*.

[38] Bessa J., Schmitz N., Hinton H. J., Schwarz K., Jegerlehner A., Bachmann M. F. (2008). Efficient induction of mucosal and systemic immune responses by virus-like particles administered intranasally: implications for vaccine design. *European Journal of Immunology*.

[39] Clements J. D., Freytag L. C. (2016). Parenteral vaccination can be an effective means of inducing protective mucosal responses. *Clinical and Vaccine Immunology*.

[40] Ball J. P., Springer M. J., Ni Y. (2017). Intranasal delivery of a bivalent norovirus vaccine formulated in an *in situ* gelling dry powder. *PLoS One*.

